# Validation of a Traditional Medicine, *Achyrocline satureioides* Infusion, for the Improvement of Mild Respiratory Infection Symptoms: A Randomized, Placebo-Controlled and Open-Label Clinical Trial

**DOI:** 10.3390/ph18060861

**Published:** 2025-06-09

**Authors:** Catherina Isdra Moszkowicz Bastos, Caroline Dani, Laura Reck Cechinel, Arthur Hipolito da Silva Neves, Fabiana Briato Rasia, Marcelo Lazzaron Lamers, Sara Elis Bianchi, Gabriela Meirelles, Paulo Valdeci Worm, Valquiria Linck Bassani, Ionara Rodrigues Siqueira

**Affiliations:** 1 Programa de Pós-Graduação em Ciências Biológicas—Farmacologia e Terapêutica, Universidade Federal do Rio Grande do Sul (UFRGS), Porto Alegre 90035-003, Rio Grande do Sul, Brazil; catherinaisdra@yahoo.com.br (C.I.M.B.); carolinedani@yahoo.com.br (C.D.); laurareck@gmail.com (L.R.C.); arthurhipolito.sneves@gmail.com (A.H.d.S.N.); fabianarasia@gmail.com (F.B.R.); marcelo.lamers@ufrgs.br (M.L.L.); 2 Programa de Pós-Graduação em Ciências Biológicas—Fisiologia, Universidade Federal do Rio Grande do Sul, Porto Alegre 90035-003, Rio Grande do Sul, Brazil; 3 Programa de Pós-Graduação em Ciências Farmacêuticas, Universidade Federal do Rio Grande do Sul, Porto Alegre 90610-000, Rio Grande do Sul, Brazil; saraelisbianchi@gmail.com (S.E.B.); gabimeirelles@gmail.com (G.M.); valquiria.bassani@ufrgs.br (V.L.B.); 4 Departamento de Cirurgia, Universidade Federal de Ciências da Saúde de Porto Alegre (UFCSPA), Porto Alegre 90050-170, Rio Grande do Sul, Brazil; paulowormnec@gmail.com

**Keywords:** cold, flu, coronavirus, macela, herbal medicine, medicinal plant, COVID-19

## Abstract

**Background/Objectives:** The need for the scientific validation of traditional and folk medicine knowledge has emerged lately. *Achyrocline satureioides* inflorescences have been widely used for the management of mild viral respiratory infection symptoms in South Brazil, Uruguay and Argentina. We intended to assess the therapeutic efficacy of a 14-day course with *A. satureioides* for mild viral respiratory infection symptoms. **Methods**: We conducted a randomized, open-label, placebo-controlled trial. Before COVID-19 (SARS-CoV-2) diagnostic tests, participants were randomly assigned to one of two experimental groups: *A. satureioides* or *Malus domestica* infusions, with instructions to use the infusions twice a day for 14 days. Our primary endpoint was the recovery time for respiratory symptoms in the overall analysis; the secondary outcomes were the recovery time for non-respiratory symptoms and for stratified analysis, taking into account the vaccination status against SARS-CoV-2 and COVID-19 infection; and the rate of symptom recovery was also evaluated. **Results**: The *A. satureioides* infusion significantly accelerated the resolution of sore throat and sneezing compared with the control group. The participants with COVID-19 who had not been vaccinated and received *A. satureioides* infusion recovered faster from sore throat, body ache, fever and cough, and showed a shorter median survival time for symptom resolution. The SARS-CoV-2-negative group that received *A. satureioides* had a faster improvement in the survival analysis of sore throat, earache and loss of appetite. **Conclusions**: Our findings support the hypothesis that *Achyrocline satureioides* inflorescence infusions may offer therapeutic benefits in the management of mild viral respiratory infections, as its administration was associated with a significantly accelerated resolution of clinical symptoms. This study was registered in the Brazilian Registry of Clinical Trials (ReBEC; registration number RBR-8g6f2rv) on 27 January 2022.

## 1. Introduction

Brazilian biodiversity accounts for over 20–22% of the global higher plant species. In addition to its rich biodiversity, Brazil also possesses a vast and culturally diverse body of traditional medicinal knowledge, particularly with regard to the use of medicinal plants (also referred to as herbal medicines) [1,2]. Although long-term traditional use may provide evidence of safety and efficacy, potential therapeutic agents, including traditional medicinal plants, need to be evaluated using current scientific approaches and methodologies to ensure safety and efficacy [1].

In this context, traditional medicinal plants have been recognized as potentially beneficial in the management of mild respiratory symptoms, including cough and fever [3]. Inflorescence infusions of the South American species, *Achyrocline satureioides* (Lam.) D. C. (Asteraceae), called “marcela” or “macela”, has been widely used for the treatment of several diseases, including respiratory infections [4]. In a clinical trial, our group published preliminary results, without a complete follow-up, of the beneficial effects of *A. satureioides* inflorescence infusions on viral respiratory infection symptoms, including those induced by SARS-CoV-2, adding evidence to the ethnopharmacological profile [5]. Interestingly, *A. satureioides* infusion improved the latency to the resolution of fever, sore throat, the respiratory symptoms of cough and dyspnea, and neurological symptoms of smell and taste dysfunctions compared with the control group (*M. domestica* infusion) [6]. This work offers an update to this preliminary report [6], describing a randomized, placebo-controlled, open-label clinical trial performed to assess the clinical efficacy of a 14-day course of an *A. satureioides* inflorescence infusion twice a day for the treatment of mild viral respiratory infection symptoms, including those caused by COVID-19, after a complete follow-up.

## 2. Results

### 2.1. Participants and Baseline Characteristics

**Figure 1:**
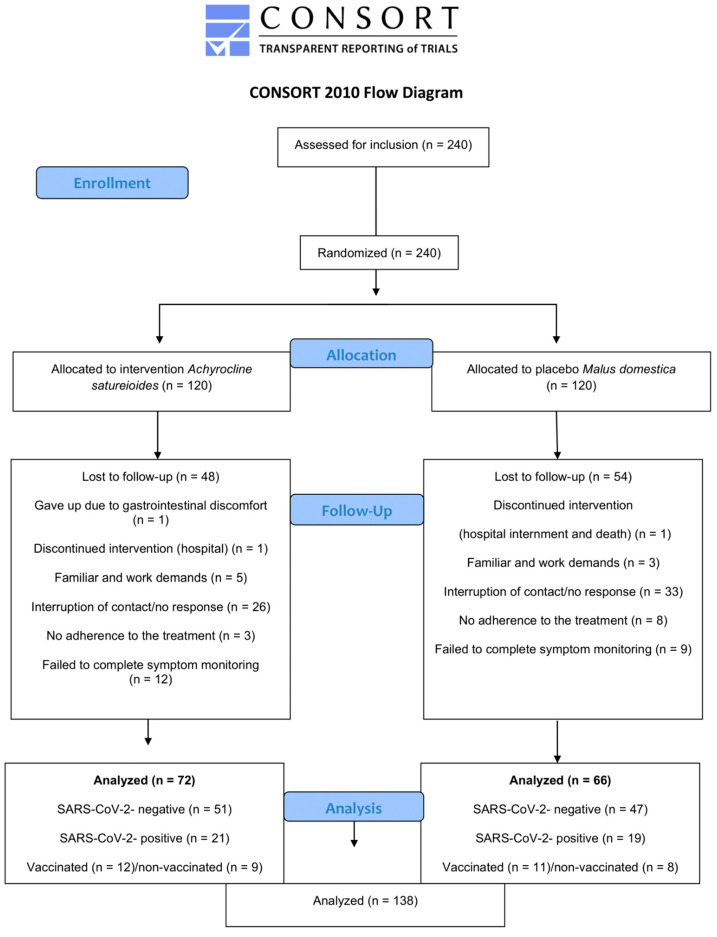
CONSORT flowchart of the randomized placebo-controlled trial to compare the effects of the *A. satureioides* infusion with the *M. domestica* infusion, showing the random assignment of participants and indicating the number of subjects who were enrolled, and additional information such as the vaccination condition, those lost to follow-up, and SARS-CoV-2-positive and SARS-CoV-2-negative distributions.

We collected data between 24 March 2021 and 6 December 2021. A total of 240 participants were assessed for eligibility and underwent randomization. Figure 1 shows the flow diagram. Of the 240 eligible participants included in the study, 138 completed symptom monitoring, 72 in the *A. satureioides* group and 66 in the *M. domestica* group. Participants withdrew during the follow-up due to family or work demands, a lack of time to prepare the infusion and fear of exposure to COVID-19. There was no significant difference in loss to follow-up between *A. satureioides* infusion and *M. domestica* infusion groups (chi square test, *p* = 0.4). The baseline demographic and clinical characteristics of the participants are reported in Table 1. Both groups were comparable in terms of the baseline characteristics.

### 2.2. Overall Analysis

**Figure 2:**
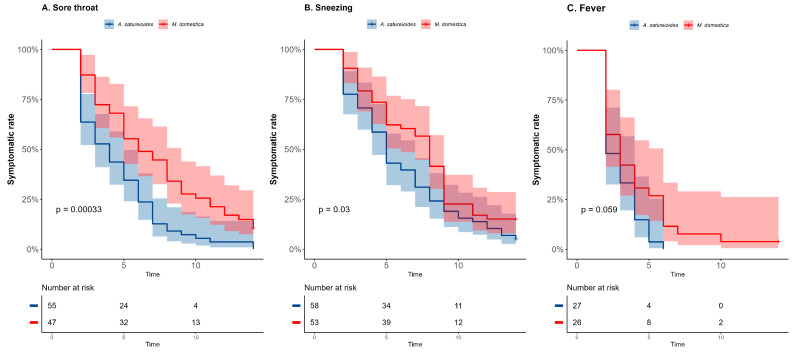
*Achyrocline satureioides* infusion affected the recovery time for mild respiratory viral infection symptoms. Kaplan–Meier curves for latency (in days) to the total recovery of clinical symptoms, sore throat (**A**), sneezing (**B**) and fever (**C**) in the overall analysis during the 14-day follow-up. The percentage of participants who achieved symptom resolution at individual time points was demonstrated for the *A. satureioides* (red) and *M. domestica* (blue) groups. The shaded areas indicate the 95% confidence intervals. The horizontal dashed line in each panel indicates the median survival time (time with 50% resolution rate of the symptom).

The *A. satureioides* infusion significantly impacted the recovery time for some respiratory symptoms, including the primary outcome, specifically sore throat and sneezing, compared with the *M. domestica* group in the overall analysis (Figure 2A,B). A shorter median time spent suffering from sore throat was observed in the *A. satureioides* group (4 days [95% CI, 2 to 5] vs. *M. domestica* group, 6 days [95% CI, 5 to 8], *p* < 0.01; HR = 2.13 [95% CI, 1.41 to 3.24], *p* < 0.01). In addition, the latency to remission of sneezing (5 days [95% CI, 4 to 7] vs. 8 days [95% CI, 5 to 9], *p* = 0.03; HR = 1.50 [95% CI, 1.01 to 2.23], *p* = 0.043) was observed.

In accordance, the rate of symptom recovery on day 8 was significantly higher in the *A. satureioides* group compared with the *M. domestica* group for sore throat (87.2% vs. 55.3%, *p* = 0.0003) and for sneezing (68.9% vs. 43.3%, *p* = 0.007). Fisher’s exact test revealed a trend for an effect of *A. satureioides* on the rate of symptom recovery for sore throat on day 14 in the overall analysis (96.3% vs. 85.1%, *p* = 0.077). The A. *satureioides* group showed 2 days (95% CI, 2 to 4) to recover from fever, while the *M. domestica* infusion group had 3 days (95% CI, 2 to 4), with a trend toward statistical significance (Figure 2C, *p* = 0.059). Consistently, the Hazard Ratio (HR) from fever was 1.71 ([95% CI, 0.95 to 3.07], *p* = 0.06). An effect of *A. satureioides* infusion on the rate of dyspnea recovery on day 8 was also observed in the overall analysis (62.1% vs. 40%, *p* = 0.046) (Supplementary Figure S1A). Although the *A. satureioides* infusion group seemed to have a faster respiratory recovery, specifically for sore throat and sneezing, there were no significant differences between the *A. satureioides* group and *M. domestica* group on cough (Supplementary Figure S1B). Furthermore, non-respiratory symptoms, such as a loss of appetite, earache and body ache, did not reach any statistical significance in the overall analysis (Supplementary Figure S1C–E).

### 2.3. Subgroup Analysis

Subgroup analyses considering the vaccination status against SARS-CoV-2 (vaccinated and non-vaccinated participants) and COVID-19 infection (SARS-CoV-2+ or SARS-CoV-2−) were performed.

**Figure 3:**
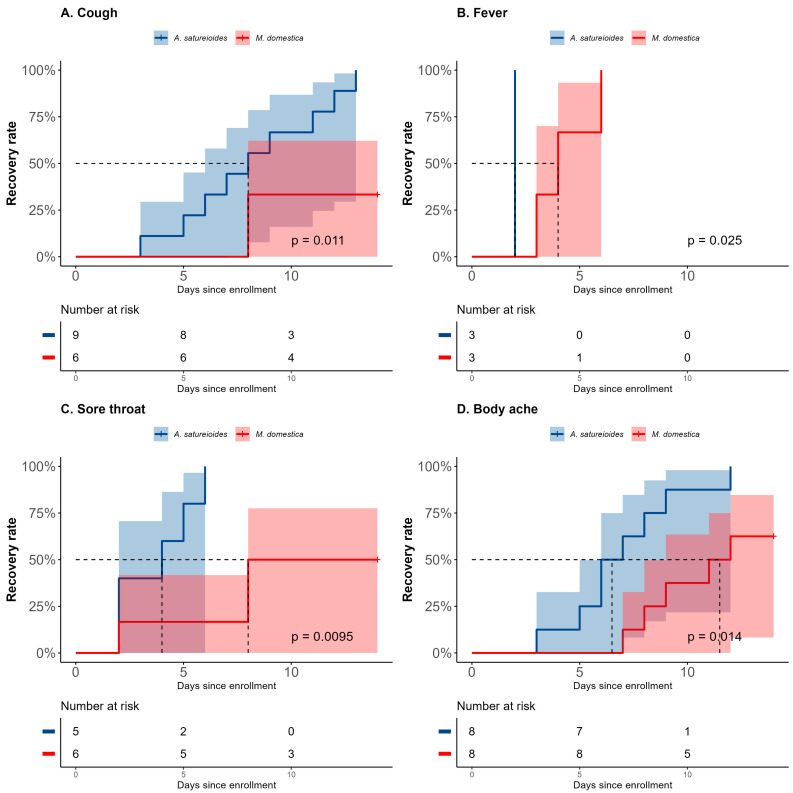
Kaplan–Meier curves for latency (in days) to the total recovery of clinical symptoms, cough (**A**), fever (**B**), sore throat (**C**) and body ache (**D**) in the non-vaccinated SARS-CoV-2-positive subgroup analysis during the 14-day follow-up. The horizontal dashed line in each panel indicates the median survival time (time with 50% resolution rate of the symptom).

In the subgroup analyses, participants who were non-vaccinated, SARS-CoV-2-positive and had received *A. satureioides* infusion had significantly fewer days with cough (8 [95% CI 3 to 12] vs. NE [8 to NE], *p* = 0.011; HR: 5.74 [95% CI: 1.19 to 27.55], *p* = 0.02; Figure 3A). In accordance, on day 14, the *A. satureioides* infusion group also had higher recovery rates when compared with *M. domestica* for cough (100% vs. 33.33%, *p* = 0.01) among those SARS-CoV-2-positive participants. *A. satureioides* infusion in the non-vaccinated SARS-CoV-2-positive subgroup induced faster resolution on fever (2 [95% CI, NE to NE] vs. 4 [95% CI, 3 to NE], *p* = 0.025) and sore throat (4 days [95% CI, 2 to not estimable, (NE)] vs. 8 days [95% CI, 2 to NE], *p* < 0.01; HR = 10.33 [95% CI, 1.15 to 92.27], *p* = 0.03) (Figure 3B,C).

A statistically significant effect of *A. satureioides* infusion was observed on the rate of sore throat recovery in non-vaccinated SARS-CoV-2-positive participants on day 8 (100% [5] vs. 16.6% [1], *p* = 0.01). In addition, the *A. satureioides* infusion improved the body ache recovery (6.5 days [95% CI, 3 to 9] vs. 11.5 days [95% CI, 7 to NE], *p* = 0.014; HR: 4 [95% CI: 1.25 to 12.73], *p* = 0.01), with a rate of recovery of 75% [6] compared with the *M. domestica* infusion group (25% [2], *p* = 0.052) (Figure 3D). There were no significant differences between the *A. satureioides* and *M. domestica* groups in the non-vaccinated SARS-CoV-2-positive participants regarding dyspnea, sneezing earache and loss of appetite (Supplementary Figure S2A–D).

However, there was a significant impact of vaccination status. For example, the vaccinated SARS-CoV-2-positive subgroup that received *M. domestica* showed approximately 7 (4–9; average = 8.2 days) days suffering with cough, while the survival analysis was unable to estimate the median value because few patients (less than 50%) achieved resolution on day 14 in the non-vaccinated ones (average = 12 days, *p* = 0.034) (Supplementary Figure S3). In addition, there were no significant differences between the *M. domestica* infusion group and the *A. satureioides* group regarding the median time to recovery for any evaluated symptoms in the vaccinated SARS-CoV-2-positive subgroup (Supplementary Figure S4A–D).

**Figure 4:**
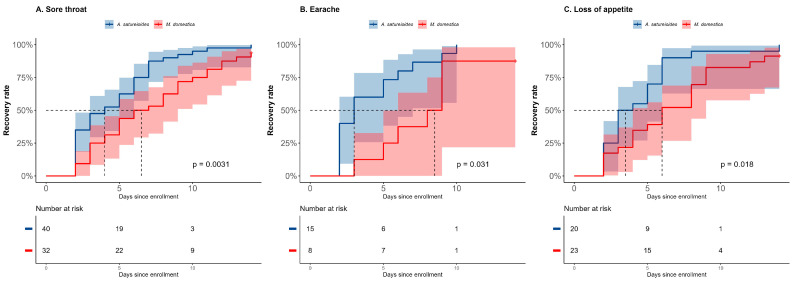
Kaplan–Meier curves for latency (in days) to the total recovery of clinical symptoms, sore throat (**A**), earache (**B**) and loss of appetite (**C**) in the SARS-CoV-2-negative subgroup analysis during the 14-day follow-up. The horizontal dashed line in each panel indicates the median survival time (time with 50% resolution rate of the symptom).

The SARS-CoV-2-negative *A. satureioides* subgroup had a significantly shorter recovery time for sore throat as well (4 days [95% CI, 2 to 6] vs. 6.5 days [95% CI, 4 to 9], *p* < 0.01; HR = 2.09 [95% CI, 1.28 to 3.42], *p* < 0.01). There was a significantly higher rate of recovery for sore throat on day 8 (87.5% vs. 53.1%, *p* = 0.001; Figure 4A).

The recovery time for earache was impacted by *A. satureioides* infusion (3 days [95% CI, 2 to 5] vs. 8.5 days [95% CI, 3 to 9], *p* = 0.031; HR: 2.49 [95% CI: 0.98 to 6.15], *p* = 0.052), and the rate of recovery on day 8 was 86.6%, while the *M. domestica* group showed 37.5% (*p* = 0.026) in the SARS-CoV-2-negative subgroup (Figure 4B). In this subgroup, the use of *A. satureioides* infusion resulted in a shorter duration of loss of appetite (3 days [95% CI, 2 to 5] vs. 6 days [95% CI, 4 to 8], HR: 2.14 [95% CI: 1.13 to 4.06], both *p* = 0.018), with a significant rate of recovery on day 8 (90% vs. 52.1%, *p* = 0.009; Figure 4C). In addition, the *A. satureioides* infusion improved the rate of dyspnea recovery on day 8 (64% vs. 36.3%; *p* = 0.037) and the sneezing recovery rate (65.8% vs. 42.1%, *p* = 0.034) compared with the control group with the *M. domestica* infusion.

### 2.4. Safety

There were no significant adverse events identified; however, one participant in the *Achyrocline satureioides* group experienced a mild adverse event, specifically stomach discomfort, which led to withdrawal from the trial.

## 3. Discussion

Our data support the hypothesis that *A. satureioides* infusions twice a day for 14 days can induce significantly faster symptom recovery and may improve the rate of recovery of respiratory infection disease symptoms, including in symptomatic patients of mild COVID-19, compared with the control group with a *Malus domestica* infusion. We found significant alleviation of several symptoms, sore throat, sneezing, cough, body ache, fever, earache and loss of appetite. Beyond the efficacy of *A. satureioides* on respiratory infection symptoms, it is possible to describe that the administration of *A. satureioides* infusions appears to be generally well-tolerated and safe.

It is important to highlight that *A. satureioides* induced 2- and 2.5-day reductions in sore throat in the overall analysis and in the SARS-CoV-2-negative group, while the *A. satureioides* SARS-CoV-2-positive group had a median recovery time of 4 days compared with the 8 days of the *M. domestica* group. The efficacy of mefenamic acid combined with standard medical care (mainly treatment with acetaminophen) compared with standard medical care and a placebo was evaluated in a prospective, randomized, double-blind, two-arm trial in ambulatory patients with COVID-19 [7]. The mefenamic acid group had 2.5-day reductions in sore throat compared with the placebo group [7]. Taken together, this can be seen as clinically meaningful by improving patient comfort, reducing school or work absenteeism and potentially decreasing healthcare utilization. This is especially relevant during viral outbreaks, when healthcare systems are under pressure since even small improvements in symptom duration may have a broader public health impact.

The *A. satureioides* infusion seems to have had superior efficacy in the non-vaccinated SARS-CoV-2-positive individuals. Briefly, the non-vaccinated SARS-CoV-2-positive patients had improvements on cough, body ache, sore throat and fever recovery by *A. satureioides* infusion. In addition, sore throat, loss of appetite, earache and dyspnea showed faster resolution in the SARS-CoV-2-negative subgroup. Although *A. satureioides* showed significant effects on sore throat and sneezing in the overall analysis, no statistically significant benefits were observed for cough and dyspnea. These findings suggest that clinical heterogeneity may explain the lack of effect in the overall analysis and reinforce the relevance of stratified interpretations. We can suppose that the non-vaccinated SARS-CoV-2-positive group was a more homogeneous group because they had this coronavirus as a pathogen, while the overall and SARS-CoV-2-negative groups had a high heterogeneity, such as rhinovirus; parainfluenza; influenza viruses; and several potential bacteria, such as *Streptococcus pyogenes* [8]. We previously concluded that these miscellaneous infections could have biased our analysis, and further studies must be conducted to evaluate the effects of *A. satureioides* on specific viruses, such as rhinovirus and influenza virus.

Sore throat is related to respiratory mucosa of the throat infection induced by several viruses, including coronavirus, rhinovirus, respiratory syncytial virus and Epstein–Barr virus, and by bacteria species, such as *Streptococcus* sp., *Haemophilus influenzae* and *Moraxella catarrhalis*; however, most sore throat cases seem to have a virus as the causative organism [9]. Although some remarkable outcomes, such as HR = 10.33 for sore throat recovery in the non-vaccinated SARS-CoV-2-positive participants, were overlapped and supported by internal consistency across impacted symptoms, we can infer a weakness of our work based on the lack of corrections for multiple comparisons (e.g., Bonferroni) and/or small subgroup sizes.

Beyond a statistical significance denoted by *p* values, we could point out the range of values estimated by 95% CIs in order to have a practical significance analysis. In the overall analysis, we observed, for example, 2 to 5 days (95% CI) suffering with sore throat in the *A. satureioides* group, while the *M. domestica* group had 5 to 8 days. Another example about the subgroup analyses, where even with the small sample size, participants who were non-vaccinated SARS-CoV-2-positive with cough and in the control group had 8 to NE days, while the *A. satureioides* group had a 95% CI of 3 to 12 days. Although a 95% CI can be a useful statistical measure, some data had very early resolution in the *A. satureioides* group, for example, fever (Figure 3B), which compromised the 95% CI estimation since all participants recovered by day 2, even without any censored observations. Consequently, due to the small sample size and lack of variability, the 95% CI for this median recovery time could not be estimated and was therefore reported as “Not Estimable” (NE).

Given the symptom profile impacted by *Achyrocline satureioides*, anti-inflammatory characteristics can be hypothesized as a primary mode of action. A hydroalcoholic extract obtained from *A. satureioides* inflorescences reduced the neutrophil infiltration and the levels of inflammatory mediators in a model of inflammation induced by subcutaneous lipopolysaccharide (LPS) injection in male Wistar rats, lending support to this idea [10]. De Souza, Basani and Schapoval [11] evaluated the anti-inflammatory effect of *A. satureioides* spray-dried and freeze-dried powders in a carrageenan-induced rat paw edema model and found significant antiedematogenic properties and a reduction in total leukocyte and polymorphonuclear cell migration in the pleural cavity.

Even though it is impossible to indicate at this moment exactly which phytocompound(s) is (are) responsible for the huge effects of the *A. satureioides* infusion, their flavonoids can be considered as relevant candidates. Di Pierro et al. [12] conducted a clinical trial in order to observe the effects of an oral quercetin supplement (500 mg) for one week in patients infected with SARS-CoV-2. Compared with the placebo groups, participants that used quercetin had a faster recovery of symptoms and tested negative earlier in the follow-up. Araújo et al. [13] showed quercetin’s ability to prevent lung injury caused by cigarette smoke using in vitro and in vivo models, where pulmonary parenchyma and lung function were protected due to the antioxidant and anti-inflammatory activities of quercetin [13]. Another potential mechanism can be raised based on in vitro findings since quercetin was able to relax the airway smooth muscle of tracheal rings from mice upon exposure to acetylcholine, similarly to methylxanthines, where quercetin inhibited the phosphodiesterase activity, a known mechanism of anti-asthmatics [14]; this could be involved with our findings on cough and dyspnea improvements induced by *A. satureioides* infusions.

In addition, the antiviral activity of quercetin may have contributed to symptom improvements; however, the antiviral mechanism of action of quercetin is not widely well understood. Quercetin is able to bind to the glycoprotein hemagglutinin of the influenza A virus, inhibiting virus entry into host cells [15]. Concerning SARS-CoV-2, quercetin binds to the angiotensin-converting enzyme 2 (ACE2) spike protein, preventing virus–host recognition and the virus entrance into the host cell [16]. An in vitro study showed that quercetin inhibits syncytium formation in cells coexpressing the viral spike protein and human ACE2 [17]. Furthermore, a molecular docking study reported that quercetin inhibits the transmembrane serine protease 2 (TMPRSS2), a crucial protease involved in the proteolytic cleavage of the SARS-CoV-2 spike protein, and consequently, for its activation and binding to the ACE2 receptor [18].

Additionally, quercetin prevents viral replication suppressing the activity of 3-chymotrypsin-like protease (3CLpro), a key enzyme involved in viral replication [16,19]. Considering this potential mechanism of action of isolated compounds from *Achyrocline satureioides*, the treatment regimen used here (twice a day for 14 days) was based on clinical trials that evaluated lopinavir/ritonavir (inhibitors of 3CLpro) for the management of COVID-19 [20]. In addition, the used dose (1.5 g in 150 mL of boiling water) was adopted in accordance with traditional practices in Southern Brazil and what is preconized in the Brazilian Pharmacopoeia [21].

It is relevant to point out that the early intervention, beginning at the first medical care seeking, was based on the previously described preclinical antiviral activities of *A. satureioides* [5] and the role of viral replication levels in the first week of symptoms of COVID-19 [22]. Given that COVID-19 diagnoses by RT-PCR took up to 3 days, the inclusion and intervention happened already at the first medical care seeking.

The findings on the final, all-randomized sample analysis reported here were mainly consistent with those of the preliminary report with an interim analysis [6], with a faster improvement of the symptoms induced by the *A. satureioides* infusion. It is relevant to note that this preliminary report includes data collection from participants between 24th March and 24th May of 2021; at that moment, only 18% of COVID-19 participants were vaccinated because the vaccines were not properly and widely distributed in our country. During the second phase of data collection, which happened between 26th July and 23rd November, all the included COVID-19 participants were already vaccinated, which resulted in a total of 52.4% of the vaccinated participants with COVID-19 in the final analysis. The vaccination impacted the severity of clinical outcomes, where the vaccinated SARS-CoV-2-positive subgroup, even those that received the *M. domestica* infusion, showed faster resolution, in accordance with previous findings [23]. Interestingly, we did not observe a synergistic effect between the vaccines and *A. satureioides* infusion to recovery for any evaluated symptoms in the vaccinated SARS-CoV-2-positive subgroup. A potential explanation for the lack of a synergistic effect between the vaccination and *A. satureioides* in the SARS-CoV-2-positive group was a similar protective immune response to combat the virus, and consequently, the vaccines already induced a primed immune response. Although no immunological markers (e.g., cytokine levels or antibody titers) were evaluated in this study, which limits our ability to explore this mechanistic interaction, it is recognized that some plant compounds are able to modulate humoral and cell-induced immune responses, such as those related to Th1 and Th2 [24].

Although the high efficacy rate of vaccines against SARS-CoV-2-induced infections is unarguable, new mutations in the SARS-CoV-2 genome have been described and some variants can reduce the effects of antibodies generated by both infection and vaccination [25], which can be associated with reduced vaccine efficacy and increased transmissibility and risk of reinfection [26], bringing the need for new effective approaches for COVID-19, including those based on traditional medicine [27].

We cannot disregard the potential biological properties of *M. domestica* tea; however, most studies have attributed reduced risks of cancer, cardiovascular disease and diabetes. It is relevant to mention that polyphenolic and phytosterol compounds have been related to the properties of *M. domestica*. Patocka et al. (2020) raised that β-sitosterol, which has been found in apples, shows expectorant action, anti-asthma and antitussive activities [28]. All of them are relevant to the clinical outcomes of our clinical trial. Actually, these biological properties of apples can be related to different findings between experimental and control groups using the overall analysis or SARS-CoV-2-negative group.

Our study had potential limitations. First, our findings are based on an open-label trial since it was impossible to conduct this trial with a double-blind design because of the differences in taste, smell and appearance of the infusions. However, to avoid or reduce this bias, the participants received information about the project, including in the written informed consent form, as the central aim was to compare the effects of plants containing phenolic compounds: “apple” and “marcela” [6]. Although an intention-to-treat (ITT) analysis would be recommended, there was a biased exclusion of noncompliance due to the high dropout rate, which brought potential implications. The analysis presented in the manuscript follows a per-protocol approach because there were equal numbers of patients in the groups despite the loss of participants that occurred due to family or work demands, a lack of time to prepare the infusion and a fear of exposure to COVID-19, without any reported side effects; consequently, we did not observe losses related to the intervention *per se*, which would exclude the validity of a PP analysis.

As highlighted by Scheim et al. (2023), this approach is appropriate in contexts with high rates of non-compliance, as it allows for a more accurate estimation of the treatment effect among those who actually followed the protocol [29]. In our study, many participants who initially enrolled did not return for a follow-up. Therefore, the PP analysis was considered the most suitable strategy to reflect the actual effectiveness of the intervention among the adherent participants, as an intention-to-treat analysis could potentially underestimate the treatment effects. In addition, our results cannot be extrapolated to younger participants; however, older patients may face more severe courses of viral respiratory infections.

## 4. Materials and Methods

### 4.1. Ethical Considerations

The study was approved by the Ethics Committee of Universidade Federal do Rio Grande do Sul (approval number 4.514.201, approval date: [21 January 2021]). It was registered at the Brazilian Registry of Clinical Trials (ReBEC; registration number RBR-8g6f2rv) on 27 January 2022.

### 4.2. Trial Design and Randomization

As previously described with our preliminary data [6], this was a phase 2, randomized, open-label, placebo-controlled trial to compare the impact of infusions of *A. satureioides* inflorescences with dehydrated apple tea infusion using *Malus domestica*. The apple infusion was used as a control, considering its polyphenol level [30,31]. Although *M. domestica* contains low levels of polyphenols, it was selected for its safety, availability and acceptability by participants.

It is necessary to clarify that it was impossible to design a blind study on the *A. satureioides* infusion (tea) because of its distinctive flavor. Even though the trial was open-label due to the distinct sensory characteristics of the infusions, the participants were informed, where the informed consent form stated that the aim of our study was to study medicinal plants, specifically teas made from plants rich in compounds called polyphenols or phenolic compounds, to investigate whether they could help in the treatment of respiratory infections caused by viruses, without mentioning the assignment of an experimental or control group. We believe that this description, as included in the informed consent form, prevented bias and we inferred that it provided efficient masking, reinforcing the validity of the study and minimizing subjective and experimental biases.

Eligible patients were older than 18 years old and suffering with viral respiratory infection symptoms, such as fever, cough and/or fatigue. The exclusion criteria were severe cases (need of hospitalization at the first medical care seeking), *A. satureioides* or apple intolerance for either sex, and women who were pregnant or with reproductive potential (without any contraceptive use). The participants asked for medical care at the Municipal Screening Unit (UMT) for COVID-19 in Igrejinha (29.5734° S, 50.7925° W) and the Primary Healthcare Units of “Grupo Hospitalar Conceição” of Porto Alegre (30.0100° S, 51.0928° W), all of which were located in Rio Grande do Sul State, Brazil. The sample size determination required for the complete clinical trial has been thoroughly described [6]. Briefly, the sample size was calculated using G*Power software, version 3.1.9.2, with 80% power and a 0.05 significance level (one-tailed), based on an expected difference of 40% in IL-6 levels. Based on these parameters, 105 participants per group would be required. Anticipating a dropout rate of 10–15%, we adjusted the sample size to 120 participants per group [6]. A total of 240 eligible patients were enrolled.

Eligible patients were invited and were verbally informed about the study. After reviewing all of the research information and agreeing to participate, they read and signed an informed consent form. All efforts were made to ensure accurate results, as well as the integrity and confidentiality of the study participants.

Block randomization with varying block sizes was performed using the virtual platform www.randomization.com (24 February 2021) [6]. We used a block randomization for each Health Unit to maintain a balance of groups assignment and reduce the potential for selection bias. No stratification factors were used during the randomization; however, stratified subgroup analyses were conducted for the SARS-CoV-2 status and vaccination status.

Packages that contained the inflorescences or the dehydrated apples were available to participants immediately after the randomization.

### 4.3. Plant Material and Intervention

*A. satureioides* inflorescences and dehydrated apple (*M. domestica*) were provided by the Kampo de Ervas (Turvo, Paraná, Brazil), with organic certification by ECOCERT^®^. The availability of genetic resources was reported to the National System, SISGEN (A928BF2).

The quality control, phytochemical and microbiological characterization of the *A. satureioides* and *M. domestica* infusions were previously described in detail in our preliminary report [6]. This study described the presence of quercetin, luteolin, 3-O-methylquercetin and achyrobichalcone in the *A. satureioides* infusion using high-performance liquid chromatography (HPLC) [6].

The participants were instructed on the infusion preparation in accordance with the Brazilian Pharmacopoeia 6th edition, adding 1.5 g of the received plant material to 150 mL of boiling water with infusion for 15 min, and on the infusion use pattern, which was twice a day for 2 weeks [6]. The preparation steps were demonstrated in person and reinforced with an illustrated infographic to ensure adherence and minimize performance bias. Considering that there are no differences in the sociodemographic and clinical characteristics of the participants between the experimental and control groups, we could exclude a selection bias (and also confounding bias) with an efficient randomization and clear inclusion/exclusion criteria.

### 4.4. Assessment

Sociodemographic and additional clinical characteristics, such as comorbid conditions, allergies and prescribed drugs, were collected with a 31-item questionnaire at the first medical care seeking (baseline). Signs and symptoms were collected at least once a day using a semi-structured questionnaire, which was available with remote monitoring approaches to be filled online (https://forms.gle/u6agfX3UMtNQSfbw7, 14 April 2021), using an app, by telephone or with paper. All participants used exactly the same questionnaire with standardized instructions. Symptom tracking was reinforced by regular follow-ups via phone or WhatsApp by a trained researcher to clarify responses and verify adherence. The medical records were also checked on signs and symptoms. Nasopharyngeal swabs were used for the SARS-CoV-2 detection (RT-PCR) according to the guidelines for the collection and transportation of the Central Laboratory of Public Health (LACEN/Porto Alegre, Rio Grande do Sul, Brazil) following the United States Centers for Disease Control and Prevention (CDC) diagnostic panel. The vaccination status was reported by the participants and checked in their medical records. Blood samples and saliva were collected before and after the intervention.

### 4.5. Endpoints

Our primary endpoint was the recovery time for respiratory symptoms (sore throat, dyspnea, sneezing and cough), defined as the duration (number of days) from randomization to the first day free of symptoms. Secondary outcomes were the recovery time for non-respiratory symptoms (fever, body ache, earache and loss of appetite) and the recovery time for all the studied symptoms considering vaccination status against SARS-CoV-2 (vaccinated and non-vaccinated participants) and COVID-19 infection (SARS-CoV-2+ or SARS-CoV-2−). In addition, the rate of symptom recovery in the overall and stratified analyses on days 8 and 14 after enrollment was also evaluated.

### 4.6. Statistical Analysis

We performed an overall analysis that compared all the participants in the intervention group with the control group and then stratified the subgroup analysis by considering the adjusted analysis with COVID-19 infection (SARS-CoV-2+ or SARS-CoV-2−) and vaccination status against SARS-CoV-2 (vaccinated and non-vaccinated participants). After receiving the RT-PCR results, approximately 30% of the participants had COVID-19. In addition, considering *A. satureioides* can act against different pathogens related to upper respiratory tract infections [12,19,32,33,34,35], we evaluated the SARS-CoV-2-negative subgroup. The SARS-CoV-2+ participants were also stratified according to their SARS-CoV-2 vaccination status (vaccinated and non-vaccinated participants) since this was associated with severity and progression of COVID-19 symptoms [23]. The analysis followed a “per-protocol” (PP) approach. Categorical variables were described as percentages, and continuous variables were described as the mean (standard difference [SD]) and median (interquartile range [IQR]). To investigate the primary outcome, the log-rank test was employed to compare the median survival time to symptom recovery between the groups. The log-rank test compares the time to event endpoints; in our study the event was symptom recovery. The patients who did not recover were censored on day 14. The Cox regression was used to calculate the Hazard Ratios (HRs). The recovery rate was expressed as the percentage of participants self-reporting symptom absence on days 8 and 14 and compared between the groups with the chi-square test. A 95% confidence interval and significance level of 0.05 were used. The software Rstudio version 4.2.2 was used. The survival analysis was conducted using the survival and survminer packages [36,37].

## 5. Conclusions

Our findings suggest that *A. satureioides* inflorescence infusion twice a day may provide benefits in the management of mild viral respiratory infection symptoms, including those caused by COVID-19, because it consistently improved the rate of symptom recovery and resulted in significantly faster recovery. Our findings could justify the widespread use of *A. satureioides* infusion as an adjuvant therapy for managing viral respiratory tract infections symptoms. Further investigation is needed for clinical validation and better understanding of the infusion mode of action.

## Figures and Tables

**Table 1:** Sociodemographic and clinical characteristics at the baseline.

	All Participants (*n* = 240)	*Achyrocline satureioides* Infusion (*n* = 120)	*Malus domestica* Infusion (*n* = 120)
Age (years, mean ± SD)	40.17 ± 14.41	41.45 ± 15.07	38.88 ± 13.71
Male *n* (%)	104 (43.51%)	50 (41.67%)	54 (45%)
Female	136 (56.67%)	70 (58.33%)	66 (55%)
Ethnicity, *n* (%)			
White	177 (73.75%)	90 (75%)	87 (72.50%)
Pardo	46 (19.17%)	22 (18.33%)	24 (20%)
Black	13 (5.42%)	6 (5.00%)	7 (5.83%)
Not provided	4 (1.67%)	2 (1.67%)	2 (1.67%)
Education Level, *n* (%)			
Elementary school (incomplete)	59 (24.58%)	32 (26.67%)	27 (22.50%)
Elementary school	41 (17.08%)	17 (14.17%)	24 (20.00%)
High school (incomplete)	14 (5.83%)	6 (5.00%)	12 (10.00%)
High school	74 (30.83%)	42 (35.00%)	32 (26.67%)
Graduation (incomplete)	15 (6.25%)	6 (5.00%)	9 (7.50%)
Graduated	32 (13.33%)	15 (12.50%)	13 (10.83%)
Not provided	5 (2.08%)	2 (1.67%)	3 (2.50%)
Comorbidities, *n* (%)			
Diabetes	27 (11.30%)	17 (14.17%)	10 (8.33%)
Hypertension	69 (28.75%)	34 (28.33%)	34 (28.33%)
Obesity (BMI > 30 kg/m^2^)	68 (28.33%)	33 (27.50%)	34 (28.33%)
Smoking	27 (11.30%)	14 (11.67%)	13 (10.83%)
Heart diseases	21 (8.79%)	15 (12.50%)	6 (5.00%)
Neoplasias	12 (5.02%)	8 (6.67%)	4 (3.33%)
Respiratory diseases	150 (62.50%)	78 (65.00%)	72 (60.00%)
User of Medicinal Plants, *n* (%)			
Yes	180 (75.00%)	94 (78.33%)	86 (71.67%)
No	52 (21.67%)	24 (20.00%)	28 (23.33%)
Not informed	8 (3.33%)	2 (1.67%)	6 (5.00%)

Abbreviation: BMI, body mass index.

## Data Availability

The data that support the findings of this study are available from the corresponding author upon reasonable request.

## Supplementary Materials

The following supporting information can be downloaded from https://www.mdpi.com/article/10.3390/ph18060861/s1, Figure S1: Kaplan–Meier curves for the time to recovery from (A) dyspnea, (B) cough, (C) loss of appetite, (D) earache and (E) body ache in the overall analysis. Figure S2: Kaplan–Meier curves for the time to recovery from (A) dyspnea, (B) sneezing, (C) earache and (D) loss of appetite in the non-vaccinated SARS-CoV-2-positive subgroup analysis. Figure S3: Kaplan–Meier curves for the time to recovery from cough for the patients in the Malus domestica group. Non-vaccinated SARS-CoV-2-positive and vaccinated SARS-CoV-2-positive subgroup analyses. Figure S4: Kaplan–Meier curves for the time to recovery from (A) cough, (B) fever, (C) sore throat and (D) body ache in the vaccinated SARS-CoV-2-positive subgroup analysis. Figure S5: Kaplan–Meier curves for time to recovery from (A) dyspnea, (B) cough, (C) sneezing, (D) fever and (E) body ache in the SARS-CoV-2-negative subgroup analysis.

## Abbreviations

The following abbreviations are used in this manuscript:

COVID-19	Coronavirus disease 2019
SARS-CoV-2	Severe acute respiratory syndrome coronavirus 2
*A. satureioides*	*Achyrocline satureioides*
*M. domestica*	*Malus domestica*
NE	Not possible to estimate
ReBEC	Brazilian Registry of Clinical Trial
RT-PCR	Reverse transcription polymerase chain reaction
UMT	Municipal Screening Unit
SISGEN	Sistema Nacional de Gestão do Patrimônio Genético e do Conhecimento Tradicional Associado
PP	“per-protocol” principle
ACE2	Angiotensin-converting enzyme 2
TMPRSS2	Transmembrane serine protease 2
3CLpro	3-chymotrypsin-like protease

## References

[1] Braga F.C. (2021). Brazilian Traditional Medicine: Historical Basis, Features and Potentialities for Pharmaceutical Development. J. Tradit. Chin. Med. Sci..

[2] Dutra R.C., Campos M.M., Santos A.R.S., Calixto J.B. (2016). Medicinal Plants in Brazil: Pharmacological Studies, Drug Discovery, Challenges and Perspectives. Pharmacol. Res..

[3] Ballabh B., Chaurasia O.P. (2007). Traditional Medicinal Plants of Cold Desert Ladakh—Used in Treatment of Cold, Cough and Fever. J. Ethnopharmacol..

[4] Retta D., Dellacassa E., Villamil J., Suárez S.A., Bandoni A.L. (2012). Marcela, a Promising Medicinal and Aromatic Plant from Latin America: A Review. Ind. Crops Prod..

[5] Siqueira I.R., Simões C.M.O., Bassani V.L. (2021). *Achyrocline satureioides* (Lam.) D.C. as a Potential Approach for Management of Viral Respiratory Infections. Phytother. Res..

[6] Bastos C.I.M., Dani C., Cechinel L.R., da Silva Neves A.H., Rasia F.B., Bianchi S.E., da Silveira Loss E., Lamers M.L., Meirelles G., Bassani V.L. (2023). *Achyrocline satureioides* as an Adjuvant Therapy for the Management of Mild Viral Respiratory Infections in the Context of COVID-19: Preliminary Results of a Randomized, Placebo-Controlled, and Open-Label Clinical Trial. Phytother. Res..

[7] Guzman-Esquivel J., Galvan-Salazar H.R., Guzman-Solorzano H.P., Cuevas-Velazquez A.C., Guzman-Solorzano J.A., Mokay-Ramirez K.A., Paz-Michel B.A., Murillo-Zamora E., Delgado-Enciso J., Melnikov V. (2022). Efficacy of the Use of Mefenamic Acid Combined with Standard Medical Care vs. Standard Medical Care Alone for the Treatment of COVID-19: A Randomized Double-Blind Placebo-Controlled Trial. Int. J. Mol. Med..

[8] Boschiero M.N., Duarte A., Palamim C.V.C., Alvarez A.E., Mauch R.M., Marson F.A.L. (2022). Frequency of Respiratory Pathogens Other than SARS-CoV-2 Detected during COVID-19 Testing. Diagn. Microbiol. Infect. Dis..

[9] Kenealy T. (2014). Sore Throat. BMJ Clin. Evid..

[10] Barioni E.D., Santin J.R., Machado I.D., Rodrigues S.F.D.P., Ferraz-de-Paula V., Wagner T.M., Cogliati B., Corrêa dos Santos M., Machado M.D.S., Andrade S.F.D. (2013). *Achyrocline satureioides* (Lam.) D.C. Hydroalcoholic Extract Inhibits Neutrophil Functions Related to Innate Host Defense. Evid.-Based Complement. Altern. Med..

[11] De Souza K.C.B., Bassani V.L., Schapoval E.E.S. (2007). Influence of Excipients and Technological Process on Anti-Inflammatory Activity of Quercetin and *Achyrocline satureioides* (Lam.) D.C. Extracts by Oral Route. Phytomedicine.

[12] Di Pierro F., Khan A., Iqtadar S., Mumtaz S.U., Chaudhry M.N.A., Bertuccioli A., Derosa G., Maffioli P., Togni S., Riva A. (2023). Quercetin as a Possible Complementary Agent for Early-Stage COVID-19: Concluding Results of a Randomized Clinical Trial. Front. Pharmacol..

[13] da Silva Araújo N.P., de Matos N.A., Leticia Antunes Mota S., Farias de Souza A.B., Dantas Cangussú S., Cunha Alvim de Menezes R., Silva Bezerra F. (2020). Quercetin Attenuates Acute Lung Injury Caused by Cigarette Smoke Both In Vitro and In Vivo. COPD J. Chronic Obstr. Pulm. Dis..

[14] Townsend E.A., Emala C.W. (2013). Quercetin Acutely Relaxes Airway Smooth Muscle and Potentiates β-Agonist-Induced Relaxation via Dual Phosphodiesterase Inhibition of PLCβ and PDE4. Am. J. Physiol. Lung Cell. Mol. Physiol..

[15] Wu W., Li R., Li X., He J., Jiang S., Liu S., Yang J. (2015). Quercetin as an Antiviral Agent Inhibits Influenza a Virus (IAV) Entry. Viruses.

[16] Gasmi A., Mujawdiya P.K., Lysiuk R., Shanaida M., Peana M., Gasmi Benahmed A., Beley N., Kovalska N., Bjørklund G. (2022). Quercetin in the Prevention and Treatment of Coronavirus Infections: A Focus on SARS-CoV-2. Pharmaceuticals.

[17] Roy A.V., Chan M., Banadyga L., He S., Zhu W., Chrétien M., Mbikay M. (2024). Quercetin Inhibits SARS-CoV-2 Infection and Prevents Syncytium Formation by Cells Co-Expressing the Viral Spike Protein and Human ACE2. Virol. J..

[18] Manjunathan R., Periyaswami V., Mitra K., Rosita A.S., Pandya M., Selvaraj J., Ravi L., Devarajan N., Doble M. (2022). Molecular Docking Analysis Reveals the Functional Inhibitory Effect of Genistein and Quercetin on TMPRSS2: SARS-CoV-2 Cell Entry Facilitator Spike Protein. BMC Bioinform..

[19] Chen L., Li J., Luo C., Liu H., Xu W., Chen G., Liew O.W., Zhu W., Puah C.M., Shen X. (2006). Binding Interaction of Quercetin-3-β-Galactoside and Its Synthetic Derivatives with SARS-CoV 3CLpro: Structure–Activity Relationship Studies Reveal Salient Pharmacophore Features. Bioorganic Med. Chem..

[20] Cao B., Wang Y., Wen D., Liu W., Wang J., Fan G., Ruan L., Song B., Cai Y., Wei M. (2020). A Trial of Lopinavir–Ritonavir in Adults Hospitalized with Severe COVID-19. N. Engl. J. Med..

[21] Agência Nacional de Vigilância Sanitária (ANVISA) (2011). Formulário de Fitoterápicos da Farmacopeia Brasileira.

[22] Yakoot M. (2022). Nonsignificant Trends in COVID-19 Trials: Is There a Significance?. J. Med. Virol..

[23] Tan S.Y., Teo S.P., Abdullah M.S., Chong P.L., Asli R., Mani B.I., Momin N.R., Lim A.C.A., Rahman N.A., Chong C.F. (2022). COVID-19 Symptom Duration: Associations with Age, Severity and Vaccination Status in Brunei Darussalam, 2021. West. Pac. Surveill. Response.

[24] Kumar A., Sharma A., Tirpude N.V., Padwad Y., Hallan V., Kumar S. (2022). Plant-Derived Immuno-Adjuvants in Vaccines Formulation: A Promising Avenue for Improving Vaccines Efficacy against SARS-CoV-2 Virus. Pharmacol. Rep..

[25] Chen J., Wang R., Wang M., Wei G.-W. (2020). Mutations Strengthened SARS-CoV-2 Infectivity. J. Mol. Biol..

[26] Tao K., Tzou P.L., Nouhin J., Gupta R.K., De Oliveira T., Kosakovsky Pond S.L., Fera D., Shafer R.W. (2021). The Biological and Clinical Significance of Emerging SARS-CoV-2 Variants. Nat. Rev. Genet..

[27] Rodriguez-Morales A.J., Barbosa A.N., Cimerman S. (2023). Editorial: New Therapeutic Approaches for SARS-CoV-2/COVID-19. Front. Immunol..

[28] Patocka J., Bhardwaj K., Klimova B., Nepovimova E., Wu Q., Landi M., Kuca K., Valis M., Wu W. (2020). *Malus domestica*: A Review on Nutritional Features, Chemical Composition, Traditional and Medicinal Value. Plants.

[29] Scheim D.E., Aldous C., Osimani B., Fordham E.J., Hoy W.E. (2023). When Characteristics of Clinical Trials Require Per-Protocol as Well as Intention-to-Treat Outcomes to Draw Reliable Conclusions: Three Examples. J. Clin. Med..

[30] Lima V., Melo E., Lima D. (2004). Teor de Compostos Fenólicos Totais Em Chás Brasileiros. Braz. J. Food Technol..

[31] Balsan G., Pellanda L.C., Sausen G., Galarraga T., Zaffari D., Pontin B., Portal V.L. (2019). Effect of Yerba Mate and Green Tea on Paraoxonase and Leptin Levels in Patients Affected by Overweight or Obesity and Dyslipidemia: A Randomized Clinical Trial. Nutr. J..

[32] Davis J.M., Murphy E.A., McClellan J.L., Carmichael M.D., Gangemi J.D. (2008). Quercetin Reduces Susceptibility to Influenza Infection Following Stressful Exercise. Am. J. Physiol.-Regul. Integr. Comp. Physiol..

[33] Farazuddin M., Mishra R., Jing Y., Srivastava V., Comstock A.T., Sajjan U.S. (2018). Quercetin Prevents Rhinovirus-Induced Progression of Lung Disease in Mice with COPD Phenotype. PLoS ONE.

[34] Ganesan S., Faris A.N., Comstock A.T., Wang Q., Nanua S., Hershenson M.B., Sajjan U.S. (2012). Quercetin Inhibits Rhinovirus Replication In Vitro and In Vivo. Antivir. Res..

[35] Uchide N., Toyoda H. (2011). Antioxidant Therapy as a Potential Approach to Severe Influenza-Associated Complications. Molecules.

[36] Therneau T.M. (2023). A Package for Survival Analysis in R..

[37] Kassambara A., Kosinski M. (2021). Survminer: Drawing Survival Curves Using ‘ggplot2’.

