# Supplementary material for: Validation of a Traditional Medicine, Achyrocline satureioides Infusion, for the Improvement of Mild Respiratory Infection Symptoms: A Randomized, Placebo-Controlled and Open-Label Clinical Trial
Source: Pharmaceuticals (Basel). 2025 Jun 9;18(6):861. doi: 10.3390/ph18060861 (PMC12196206; doi:10.3390/ph18060861)
Supplement: Supplementary file 1 [file pharmaceuticals-18-00861-s001.zip › pharmaceuticals-3621066-supplementary.pdf]

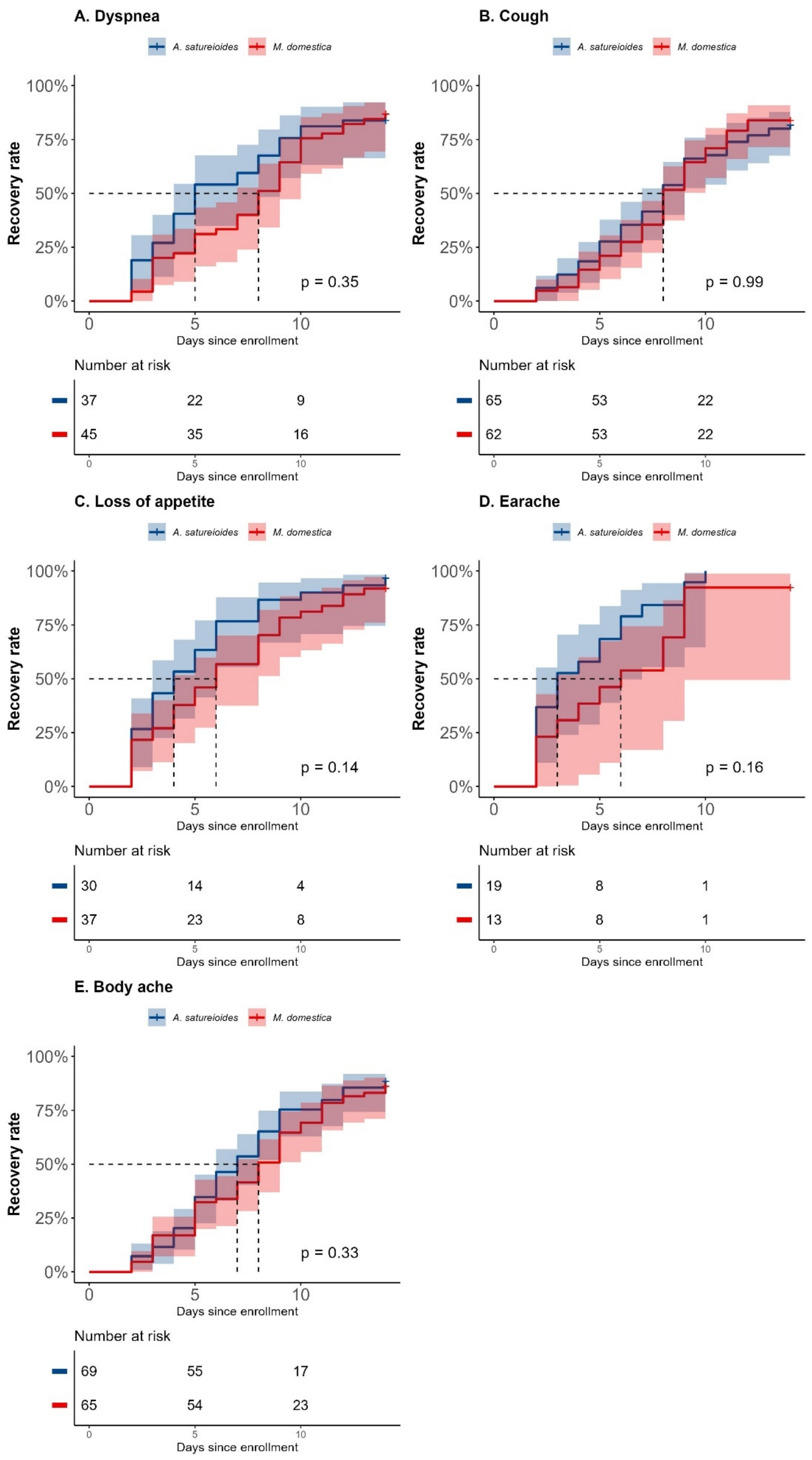

Figure S1. Kaplan-Meier curves for time to recovery from (A) Dyspnea, (B) Cough, (C) Loss of appetite, (D) Earache and (E) Body ache in the overall analysis. The percentage of participants who achieved symptom resolution at individual time points was

demonstrated for the *A. satureioides* (red) and the *M. domestica* groups (blue). The shaded areas indicate the 95% confidence intervals. The horizontal dashed line in each panel indicates the median survival time (time with 50% resolution rate of the symptom).

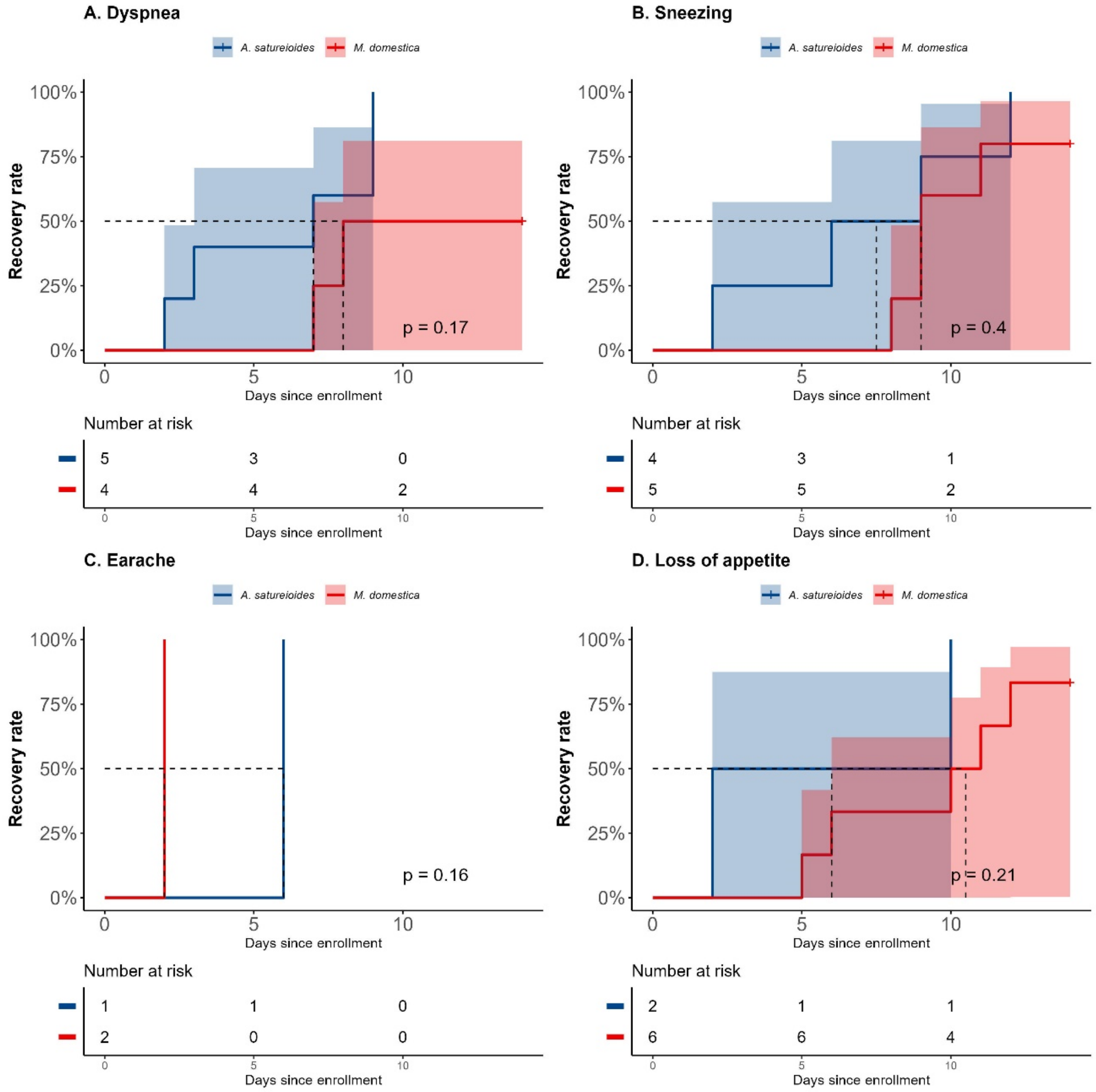

Figure S2. Kaplan-Meier curves for time to recovery from (A) Dyspnea, (B) Sneezing, (C) Earache and (D) Loss of appetite in the non-vaccinated SARS-CoV-2-positive subgroup analysis. The horizontal dashed line in each panel indicates the median survival time (time with 50% resolution rate of the symptom).

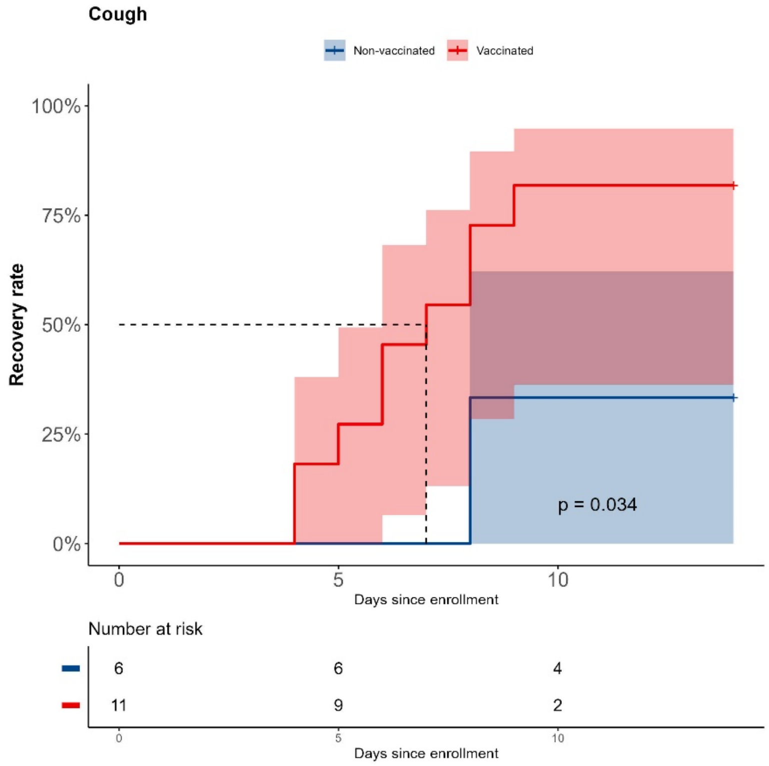

Figure S3. Kaplan-Meier curves for time to recovery from cough for patients in the *Malus domestica* group. Non-vaccinated SARS-CoV-2-positive and vaccinated SARS-CoV-2-positive subgroup analysis. The horizontal dashed line in each panel indicates the median survival time (time with 50% resolution rate of the symptom).

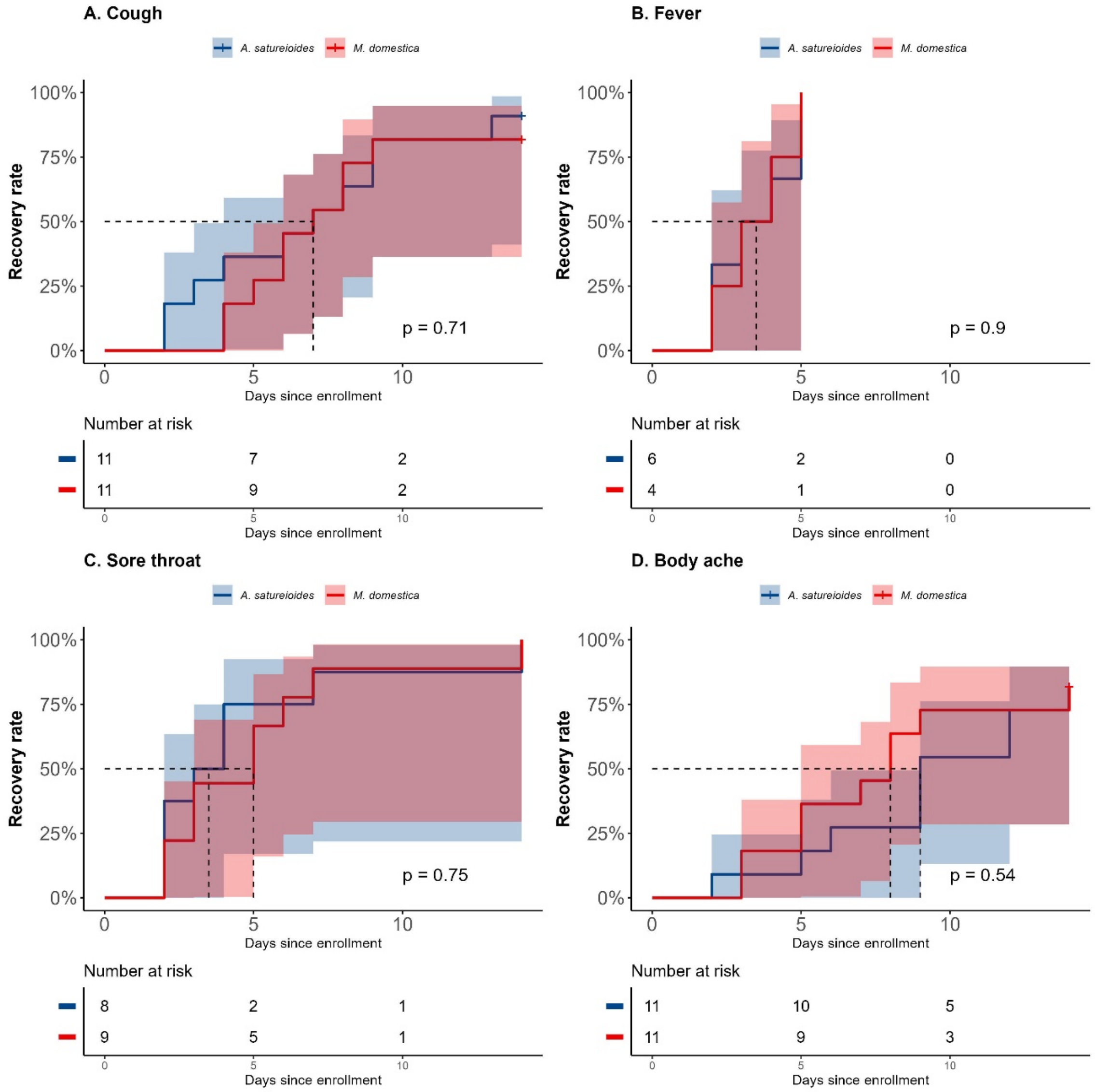

Figure S4. Kaplan-Meier curves for time to recovery from (A) Cough, (B) Fever, (C) Sore throat and (D) Body ache in the vaccinated SARS-CoV-2-positive subgroup analysis. The horizontal dashed line in each panel indicates the median survival time (time with 50% resolution rate of the symptom).

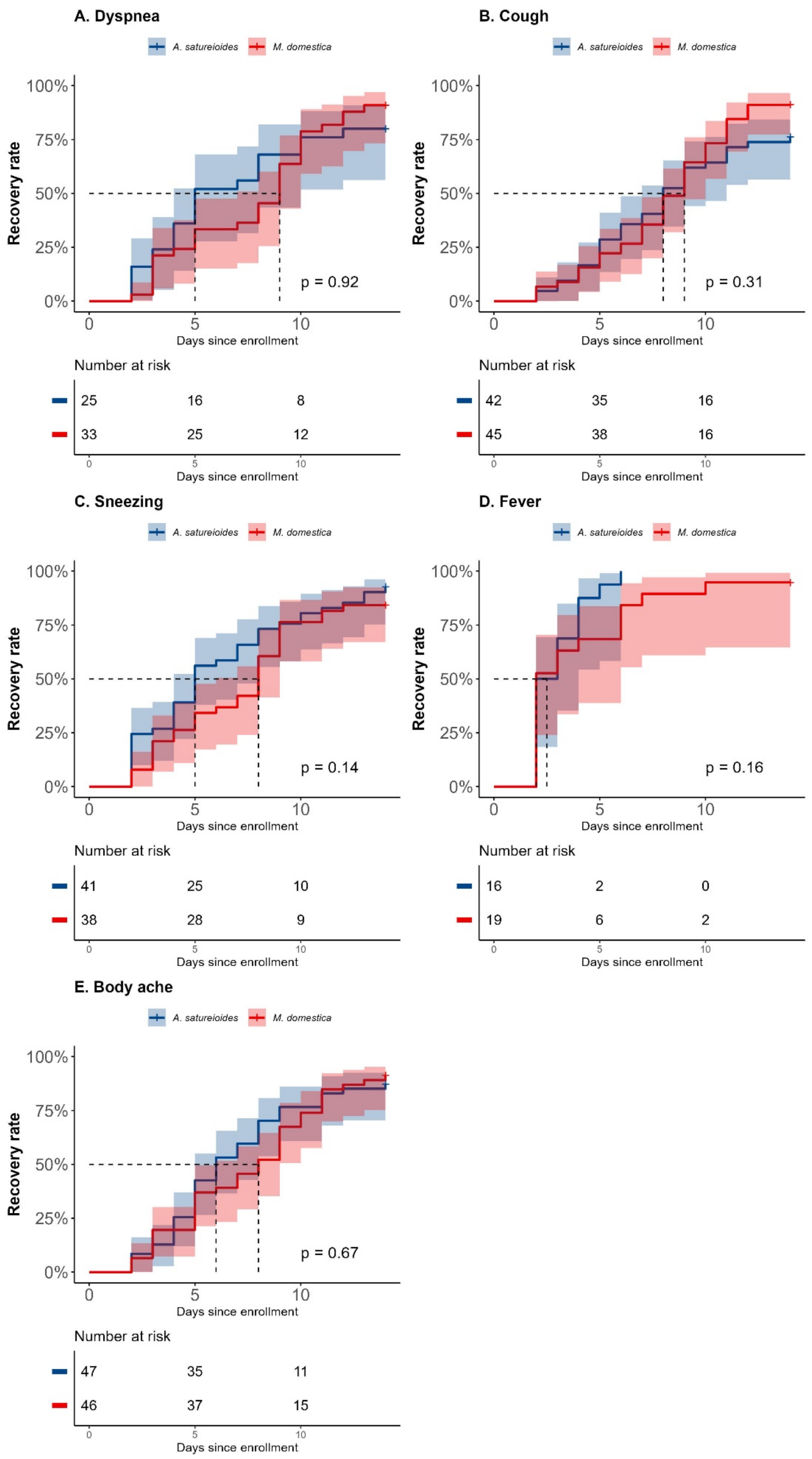

Figure S5. Kaplan-Meier curves for time to recovery from (A) Dyspnea, (B) Cough, (C) Sneezing, (D) Fever and (E) Body ache in the SARS-CoV-2-negative subgroup analysis. The horizontal

dashed line in each panel indicates the median survival time (time with 50% resolution rate of the symptom).
